# Supplementary material for: Social and anxiety-like behaviors contribute to nicotine self-administration in adolescent outbred rats
Source: Sci Rep. 2018 Dec 24;8:18069. doi: 10.1038/s41598-018-36263-w (PMC6305389; doi:10.1038/s41598-018-36263-w)
Supplement: Supplementary file 1 — Supplementary Figures and Tables [file 41598_2018_36263_MOESM1_ESM.pdf]

Social and anxiety-like behaviors contribute to nicotine self-administration in adolescent outbred rats

Tengfei Wang [1,+], Wenyan Han [1,+], Apurva S. Chitre[2], Oksana Polesskaya[2], Leah C. Solberg Woods[3], Abraham A. Palmer[2, 4], and Hao Chen[1*]

[1]Department of Pharmacology, University of Tennessee Health Science Center, Memphis, TN 38103 USA
[2]Department of Psychiatry, University of California, San Diego, La Jolla, CA 92093
[3]Department of Internal Medicine, Section on Molecular Medicine, Wake Forest School of Medicine, Winston-Salem, NC 27157
[4]Institute for Genomic Medicine, University of California, San Diego, La Jolla, CA 92093
[*]hchen@uthsc.edu
[+]These authors contributed equally to this work

Supplementary Info

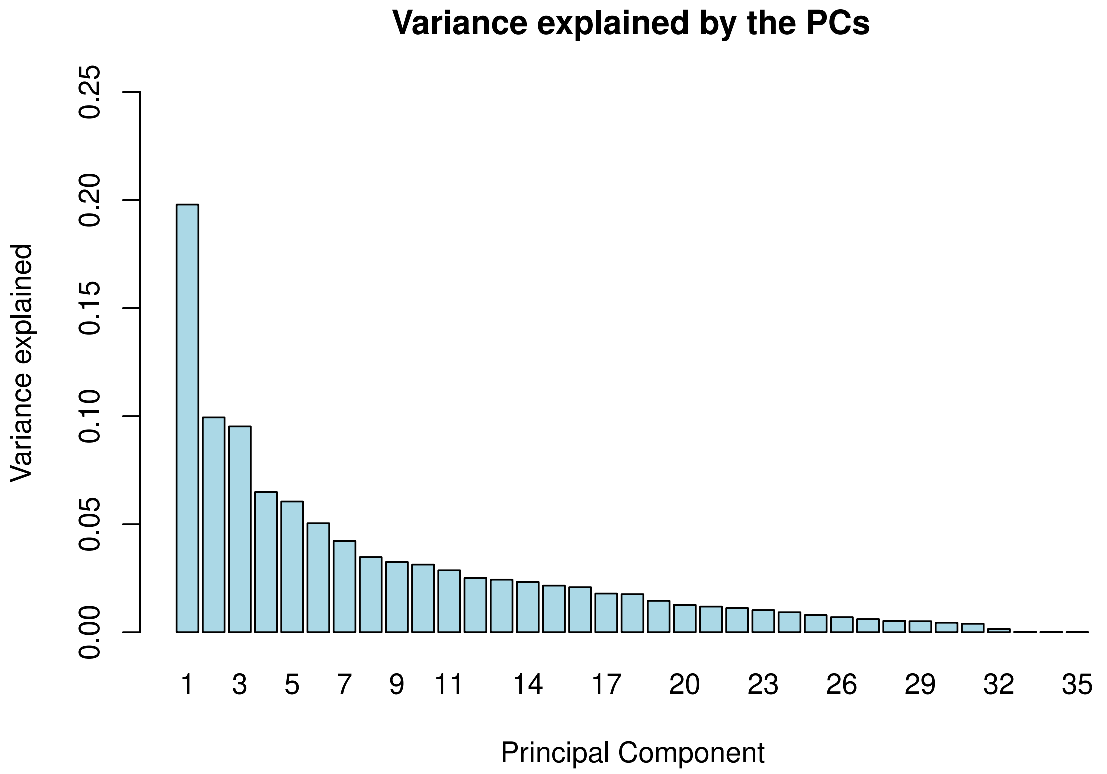

Figure S1. **Principal component analysis on 35 measures obtained from 6 behavioral tests**. The amount of variance explained by the orthogonal components were plotted.

Table S1. Open field correlations

Var1	Var2	r	p
OF Cntr Durt	OF Dist to Cntr, Mean	-0.66	$< 1e\text{-}04$
OF Cntr Durt	OF Dist to Cntr, Total	-0.58	$< 1e\text{-}04$
OF Cntr Durt	OF Total Dist	0.54	$< 1e\text{-}04$
OF Cntr Freq	OF Cntr Durt	0.77	$< 1e\text{-}04$
OF Cntr Freq	OF Dist to Cntr, Mean	-0.67	$< 1e\text{-}04$
OF Cntr Freq	OF Dist to Cntr, Total	-0.58	$< 1e\text{-}04$
OF Cntr Freq	OF Total Dist	0.72	$< 1e\text{-}04$
OF Cntr Late	OF Cntr Durt	-0.14	$< 1e\text{-}04$
OF Cntr Late	OF Cntr Freq	-0.18	$< 1e\text{-}04$
OF Cntr Late	OF Dist to Cntr, Mean	0.17	$< 1e\text{-}04$
OF Cntr Late	OF Dist to Cntr, Total	0.15	$< 1e\text{-}04$
OF Cntr Late	OF Total Dist	-0.14	$< 1e\text{-}04$
OF Dist to Cntr, Mean	OF Total Dist	-0.58	$< 1e\text{-}04$
OF Dist to Cntr, Total	OF Dist to Cntr, Mean	0.91	$< 1e\text{-}04$
OF Dist to Cntr, Total	OF Total Dist	-0.50	$< 1e\text{-}04$

Table S2. Novel object interaction correlations

Var1	Var2	r	p
NO Cntr Durt	NO Dist to Cntr, Mean	-0.85	$< 1e\text{-}04$
NO Cntr Durt	NO Dist to Cntr, Total	-0.85	$< 1e\text{-}04$
NO Cntr Durt	NO Total Dist	0.49	$< 1e\text{-}04$
NO Cntr Freq	NO Cntr Durt	0.52	$< 1e\text{-}04$
NO Cntr Freq	NO Dist to Cntr, Mean	-0.60	$< 1e\text{-}04$
NO Cntr Freq	NO Dist to Cntr, Total	-0.60	$< 1e\text{-}04$
NO Cntr Freq	NO Total Dist	0.69	$< 1e\text{-}04$
NO Cntr Late	NO Cntr Durt	-0.29	$< 1e\text{-}04$
NO Cntr Late	NO Cntr Freq	-0.30	$< 1e\text{-}04$
NO Cntr Late	NO Dist to Cntr, Mean	0.35	$< 1e\text{-}04$
NO Cntr Late	NO Dist to Cntr, Total	0.34	$< 1e\text{-}04$
NO Cntr Late	NO Total Dist	-0.37	$< 1e\text{-}04$
NO Dist to Cntr, Mean	NO Total Dist	-0.63	$< 1e\text{-}04$
NO Dist to Cntr, Total	NO Dist to Cntr, Mean	0.99	$< 1e\text{-}04$
NO Dist to Cntr, Total	NO Total Dist	-0.62	$< 1e\text{-}04$

Table S3. Social interaction correlations

Var1	Var2	r	p
SI Dist to Obj Zone, Mean	SI Total Dist	-0.16	< 1e-04
SI Dist to Obj Zone, Total	SI Dist to Obj Zone, Mean	0.99	< 1e-04
SI Dist to Obj Zone, Total	SI Total Dist	-0.15	< 1e-04
SI Dist to Soc Zone, Total	SI Dist to Soc Zone, Mean	1.00	< 1e-04
SI in Obj Zone Durt	SI Dist to Obj Zone, Mean	-0.53	< 1e-04
SI in Obj Zone Durt	SI Dist to Obj Zone, Total	-0.51	< 1e-04
SI in Obj Zone Durt	SI Dist to Soc Zone, Mean	0.33	< 1e-04
SI in Obj Zone Durt	SI Dist to Soc Zone, Total	0.33	< 1e-04
SI in Obj Zone Durt	SI Total Dist	0.20	< 1e-04
SI in Obj Zone Freq	SI Dist to Obj Zone, Mean	-0.44	< 1e-04
SI in Obj Zone Freq	SI Dist to Obj Zone, Total	-0.42	< 1e-04
SI in Obj Zone Freq	SI Dist to Soc Zone, Mean	0.28	< 1e-04
SI in Obj Zone Freq	SI Dist to Soc Zone, Total	0.27	< 1e-04
SI in Obj Zone Freq	SI in Obj Zone Durt	0.52	< 1e-04
SI in Obj Zone Freq	SI Total Dist	0.53	< 1e-04
SI in Obj Zone Late	SI Dist to Obj Zone, Mean	0.15	< 1e-04
SI in Obj Zone Late	SI Dist to Obj Zone, Total	0.15	< 1e-04
SI in Obj Zone Late	SI in Obj Zone Durt	-0.09	0.01627
SI in Obj Zone Late	SI in Obj Zone Freq	-0.21	< 1e-04
SI in Obj Zone Late	SI Total Dist	-0.22	< 1e-04
SI in Soc Zone Durt	SI Dist to Obj Zone, Mean	-0.15	< 1e-04
SI in Soc Zone Durt	SI Dist to Obj Zone, Total	-0.18	< 1e-04
SI in Soc Zone Durt	SI Dist to Soc Zone, Mean	-0.72	< 1e-04
SI in Soc Zone Durt	SI Dist to Soc Zone, Total	-0.72	< 1e-04
SI in Soc Zone Durt	SI in Obj Zone Durt	-0.31	< 1e-04
SI in Soc Zone Durt	SI in Obj Zone Freq	-0.30	< 1e-04
SI in Soc Zone Freq	SI Dist to Obj Zone, Mean	-0.12	0.00116
SI in Soc Zone Freq	SI Dist to Obj Zone, Total	-0.11	0.00292
SI in Soc Zone Freq	SI Dist to Soc Zone, Mean	-0.08	0.03043
SI in Soc Zone Freq	SI Dist to Soc Zone, Total	-0.08	0.02623
SI in Soc Zone Freq	SI in Obj Zone Freq	0.39	< 1e-04
SI in Soc Zone Freq	SI in Obj Zone Late	-0.15	< 1e-04
SI in Soc Zone Freq	SI Total Dist	0.55	< 1e-04
SI in Soc Zone Late	SI Dist to Soc Zone, Mean	0.09	0.01790
SI in Soc Zone Late	SI Dist to Soc Zone, Total	0.09	0.01657
SI in Soc Zone Late	SI in Obj Zone Late	0.26	< 1e-04
SI in Soc Zone Late	SI in Soc Zone Durt	-0.11	0.00175
SI in Soc Zone Late	SI in Soc Zone Freq	-0.11	0.00233
SI in Soc Zone Late	SI Total Dist	-0.10	0.00711

Table S4. Elevated plus maze correlations

Var1	Var2	r	p
EPM Close Durt	EPM Open Durt	-0.81	$< 1e-04$
EPM Close Durt	EPM Open Freq	-0.63	$< 1e-04$
EPM Close Durt	EPM Open Late	0.22	$< 1e-04$
EPM Close Durt	EPM Total Dist	-0.62	$< 1e-04$
EPM Close Freq	EPM Close Durt	-0.28	$< 1e-04$
EPM Close Freq	EPM Open Durt	0.18	$< 1e-04$
EPM Close Freq	EPM Open Freq	0.35	$< 1e-04$
EPM Close Freq	EPM Total Dist	0.55	$< 1e-04$
EPM Close Late	EPM Close Freq	-0.12	0.00066
EPM Close Late	EPM Open Freq	-0.10	0.00639
EPM Close Late	EPM Open Late	0.07	0.04691
EPM Close Late	EPM Total Dist	-0.17	$< 1e-04$
EPM Cntr Durt	EPM Close Durt	-0.30	$< 1e-04$
EPM Cntr Durt	EPM Close Freq	0.09	0.00876
EPM Cntr Durt	EPM Close Late	-0.09	0.01426
EPM Cntr Durt	EPM Open Durt	0.32	$< 1e-04$
EPM Cntr Durt	EPM Open Freq	0.28	$< 1e-04$
EPM Cntr Durt	EPM Open Late	-0.10	0.00507
EPM Cntr Durt	EPM Total Dist	0.22	$< 1e-04$
EPM Cntr Freq	EPM Close Durt	-0.55	$< 1e-04$
EPM Cntr Freq	EPM Close Freq	0.66	$< 1e-04$
EPM Cntr Freq	EPM Close Late	-0.14	$< 1e-04$
EPM Cntr Freq	EPM Cntr Durt	0.31	$< 1e-04$
EPM Cntr Freq	EPM Open Durt	0.47	$< 1e-04$
EPM Cntr Freq	EPM Open Freq	0.67	$< 1e-04$
EPM Cntr Freq	EPM Open Late	-0.14	$< 1e-04$
EPM Cntr Freq	EPM Total Dist	0.68	$< 1e-04$
EPM Cntr Late	EPM Close Durt	0.20	$< 1e-04$
EPM Cntr Late	EPM Close Freq	-0.15	$< 1e-04$
EPM Cntr Late	EPM Close Late	0.60	$< 1e-04$
EPM Cntr Late	EPM Cntr Durt	-0.19	$< 1e-04$
EPM Cntr Late	EPM Cntr Freq	-0.23	$< 1e-04$
EPM Cntr Late	EPM Open Durt	-0.17	$< 1e-04$
EPM Cntr Late	EPM Open Freq	-0.19	$< 1e-04$
EPM Cntr Late	EPM Open Late	0.26	$< 1e-04$
EPM Cntr Late	EPM Total Dist	-0.25	$< 1e-04$
EPM Open Durt	EPM Total Dist	0.63	$< 1e-04$
EPM Open Freq	EPM Open Durt	0.67	$< 1e-04$
EPM Open Freq	EPM Total Dist	0.64	$< 1e-04$
EPM Open Late	EPM Open Durt	-0.21	$< 1e-04$
EPM Open Late	EPM Open Freq	-0.23	$< 1e-04$
EPM Open Late	EPM Total Dist	-0.18	$< 1e-04$

Table S5. Marble bury correlation

Var1	Var2	r	p
MB. Dig Late	MB.Num Left	0.17	$< 1e-04$

Table S6. Cross behavioral test correlations, $r > 0.2$

Var1	Var2	r	p
EPM Close Durt	NO Dist to Cntr, Mean	0.22	< 1e-04
EPM Close Durt	NO Dist to Cntr, Total	0.22	< 1e-04
EPM Close Durt	NO Total Dist	-0.22	< 1e-04
EPM Cntr Durt	NO Cntr Durt	0.23	< 1e-04
EPM Cntr Durt	NO Cntr Freq	0.21	< 1e-04
EPM Cntr Durt	NO Dist to Cntr, Mean	-0.26	< 1e-04
EPM Cntr Durt	NO Dist to Cntr, Total	-0.25	< 1e-04
EPM Cntr Durt	NO Total Dist	0.21	< 1e-04
EPM Cntr Freq	NO Total Dist	0.22	< 1e-04
EPM Cntr Freq	OF Total Dist	0.22	< 1e-04
EPM Cntr Freq	SI Total Dist	0.21	< 1e-04
EPM Open Durt	NO Cntr Freq	0.22	< 1e-04
EPM Open Durt	NO Dist to Cntr, Mean	-0.23	< 1e-04
EPM Open Durt	NO Dist to Cntr, Total	-0.22	< 1e-04
EPM Open Durt	NO Total Dist	0.21	< 1e-04
EPM Open Freq	NO Total Dist	0.21	< 1e-04
EPM Open Freq	SI Total Dist	0.21	< 1e-04
EPM Total Dist	NO Cntr Freq	0.26	< 1e-04
EPM Total Dist	NO Total Dist	0.33	< 1e-04
EPM Total Dist	OF Total Dist	0.32	< 1e-04
EPM Total Dist	SI Total Dist	0.33	< 1e-04
NO Cntr Durt	OF Cntr Durt	0.27	< 1e-04
NO Cntr Durt	OF Cntr Freq	0.27	< 1e-04
NO Cntr Durt	OF Dist to Cntr, Mean	-0.31	< 1e-04
NO Cntr Durt	OF Dist to Cntr, Total	-0.26	< 1e-04
NO Cntr Durt	OF Total Dist	0.26	< 1e-04
NO Cntr Freq	OF Cntr Durt	0.22	< 1e-04
NO Cntr Freq	OF Cntr Freq	0.28	< 1e-04
NO Cntr Freq	OF Dist to Cntr, Mean	-0.26	< 1e-04
NO Cntr Freq	OF Dist to Cntr, Total	-0.21	< 1e-04
NO Cntr Freq	OF Total Dist	0.31	< 1e-04
NO Dist to Cntr, Mean	OF Cntr Durt	-0.29	< 1e-04
NO Dist to Cntr, Mean	OF Cntr Freq	-0.30	< 1e-04
NO Dist to Cntr, Mean	OF Dist to Cntr, Mean	0.33	< 1e-04
NO Dist to Cntr, Mean	OF Dist to Cntr, Total	0.28	< 1e-04
NO Dist to Cntr, Mean	OF Total Dist	-0.27	< 1e-04
NO Dist to Cntr, Total	OF Cntr Durt	-0.28	< 1e-04
NO Dist to Cntr, Total	OF Cntr Freq	-0.29	< 1e-04
NO Dist to Cntr, Total	OF Dist to Cntr, Mean	0.33	< 1e-04
NO Dist to Cntr, Total	OF Dist to Cntr, Total	0.28	< 1e-04
NO Dist to Cntr, Total	OF Total Dist	-0.26	< 1e-04
NO Total Dist	OF Cntr Durt	0.22	< 1e-04
NO Total Dist	OF Cntr Freq	0.30	< 1e-04
NO Total Dist	OF Dist to Cntr, Mean	-0.24	< 1e-04
NO Total Dist	OF Total Dist	0.39	< 1e-04
SI Dist to Obj Zone, Mean	NO Cntr Durt	-0.21	< 1e-04
SI Dist to Obj Zone, Mean	NO Dist to Cntr, Mean	0.22	< 1e-04
SI Dist to Obj Zone, Mean	NO Dist to Cntr, Total	0.22	< 1e-04
SI Dist to Obj Zone, Mean	OF Dist to Cntr, Mean	0.23	< 1e-04
SI Dist to Obj Zone, Total	NO Cntr Durt	-0.22	< 1e-04
SI Dist to Obj Zone, Total	NO Dist to Cntr, Mean	0.23	< 1e-04
SI Dist to Obj Zone, Total	NO Dist to Cntr, Total	0.23	< 1e-04
SI Dist to Obj Zone, Total	OF Dist to Cntr, Mean	0.23	< 1e-04

Table S7. Cross behavioral test correlations, $r > 0.2$, cont'd

Var1	Var2	r	p
SI in Obj Zone Durt	NO Cntr Durt	0.21	$<$ 1e-04
SI in Obj Zone Freq	NO Cntr Freq	0.21	$<$ 1e-04
SI Total Dist	NO Cntr Freq	0.21	$<$ 1e-04
SI Total Dist	NO Total Dist	0.29	$<$ 1e-04
SI Total Dist	OF Cntr Freq	0.22	$<$ 1e-04
SI Total Dist	OF Total Dist	0.37	$<$ 1e-04

Table S8. Cross behavioral tests correlations, $r < 0.2$

Var1	Var2	r	p
EPM Close Durt	NO Cntr Durt	-0.15	$<$ 1e-04
EPM Close Durt	NO Cntr Freq	-0.19	$<$ 1e-04
EPM Close Durt	NO Cntr Late	0.09	0.01271
EPM Close Durt	OF Cntr Durt	-0.11	0.00290
EPM Close Durt	OF Cntr Freq	-0.14	0.00010
EPM Close Durt	OF Dist to Cntr, Mean	0.16	$<$ 1e-04
EPM Close Durt	OF Dist to Cntr, Total	0.14	0.00010
EPM Close Durt	OF Total Dist	-0.14	$<$ 1e-04
EPM Close Durt	SI Dist to Obj Zone, Mean	0.15	$<$ 1e-04
EPM Close Durt	SI Dist to Obj Zone, Total	0.15	$<$ 1e-04
EPM Close Durt	SI in Obj Zone Durt	-0.15	$<$ 1e-04
EPM Close Durt	SI in Obj Zone Freq	-0.10	0.00521
EPM Close Durt	SI in Obj Zone Late	0.08	0.03027
EPM Close Durt	SI in Soc Zone Freq	-0.07	0.03925
EPM Close Durt	SI Total Dist	-0.17	$<$ 1e-04
EPM Close Freq	NO Cntr Freq	0.09	0.01020
EPM Close Freq	NO Total Dist	0.18	$<$ 1e-04
EPM Close Freq	OF Dist to Cntr, Total	-0.09	0.00908
EPM Close Freq	OF Total Dist	0.18	$<$ 1e-04
EPM Close Freq	SI Total Dist	0.18	$<$ 1e-04
EPM Close Late	NO Cntr Late	0.10	0.00827
EPM Cntr Durt	NO Cntr Late	-0.10	0.00548
EPM Cntr Durt	OF Cntr Durt	0.15	$<$ 1e-04
EPM Cntr Durt	OF Cntr Freq	0.15	$<$ 1e-04
EPM Cntr Durt	OF Dist to Cntr, Mean	-0.13	0.00020
EPM Cntr Durt	OF Dist to Cntr, Total	-0.10	0.00616
EPM Cntr Durt	OF Total Dist	0.10	0.00597
EPM Cntr Durt	SI Dist to Obj Zone, Mean	-0.15	$<$ 1e-04
EPM Cntr Durt	SI Dist to Obj Zone, Total	-0.15	$<$ 1e-04
EPM Cntr Durt	SI in Obj Zone Durt	0.13	0.00032
EPM Cntr Durt	SI in Obj Zone Late	-0.08	0.03089
EPM Cntr Durt	SI Total Dist	0.09	0.01272
EPM Cntr Freq	NO Cntr Durt	0.09	0.01000
EPM Cntr Freq	NO Cntr Late	-0.09	0.01049
EPM Cntr Freq	NO Dist to Cntr, Mean	-0.12	0.00061
EPM Cntr Freq	NO Dist to Cntr, Total	-0.13	0.00036
EPM Cntr Freq	OF Cntr Durt	0.08	0.02001
EPM Cntr Freq	OF Cntr Freq	0.12	0.00055
EPM Cntr Freq	OF Dist to Cntr, Mean	-0.11	0.00277
EPM Cntr Freq	OF Dist to Cntr, Total	-0.13	0.00018
EPM Cntr Freq	SI Dist to Obj Zone, Mean	-0.12	0.00096
EPM Cntr Freq	SI Dist to Obj Zone, Total	-0.13	0.00050
EPM Cntr Freq	SI in Obj Zone Durt	0.08	0.03156
EPM Cntr Freq	SI in Obj Zone Freq	0.08	0.02080
EPM Cntr Freq	SI in Obj Zone Late	-0.08	0.02191
EPM Cntr Freq	SI in Soc Zone Freq	0.07	0.04994

Table S9. Cross behavioral tests correlations, $r < 0.2$, cont'd

Var1	Var2	r	p
EPM Cntr Late	NO Cntr Freq	-0.08	0.02479
EPM Cntr Late	NO Cntr Late	0.12	0.00055
EPM Cntr Late	NO Dist to Cntr, Mean	0.08	0.03230
EPM Cntr Late	NO Dist to Cntr, Total	0.08	0.02759
EPM Cntr Late	SI Dist to Obj Zone, Mean	0.11	0.00148
EPM Cntr Late	SI Dist to Obj Zone, Total	0.11	0.00155
EPM Open Durt	NO Cntr Durt	0.18	$< 1e-04$
EPM Open Durt	NO Cntr Late	-0.10	0.00595
EPM Open Durt	OF Cntr Durt	0.11	0.00166
EPM Open Durt	OF Cntr Freq	0.16	$< 1e-04$
EPM Open Durt	OF Dist to Cntr, Mean	-0.18	$< 1e-04$
EPM Open Durt	OF Dist to Cntr, Total	-0.15	$< 1e-04$
EPM Open Durt	OF Total Dist	0.16	$< 1e-04$
EPM Open Durt	SI Dist to Obj Zone, Mean	-0.13	0.00049
EPM Open Durt	SI Dist to Obj Zone, Total	-0.13	0.00047
EPM Open Durt	SI in Obj Zone Durt	0.15	$< 1e-04$
EPM Open Durt	SI in Soc Zone Freq	0.08	0.02155
EPM Open Durt	SI Total Dist	0.19	$< 1e-04$
EPM Open Freq	NO Cntr Durt	0.15	$< 1e-04$
EPM Open Freq	NO Cntr Late	-0.11	0.00303
EPM Open Freq	NO Dist to Cntr, Mean	-0.17	$< 1e-04$
EPM Open Freq	NO Dist to Cntr, Total	-0.17	$< 1e-04$
EPM Open Freq	OF Cntr Freq	0.09	0.01790
EPM Open Freq	OF Dist to Cntr, Mean	-0.10	0.00791
EPM Open Freq	OF Dist to Cntr, Total	-0.10	0.00507
EPM Open Freq	OF Total Dist	0.19	$< 1e-04$
EPM Open Freq	SI Dist to Obj Zone, Mean	-0.17	$< 1e-04$
EPM Open Freq	SI Dist to Obj Zone, Total	-0.17	$< 1e-04$
EPM Open Freq	SI in Obj Zone Durt	0.19	$< 1e-04$
EPM Open Freq	SI in Obj Zone Freq	0.11	0.00193
EPM Open Freq	SI in Soc Zone Durt	0.08	0.03517
EPM Open Freq	SI in Soc Zone Freq	0.08	0.02038
EPM Open Late	NO Cntr Freq	-0.07	0.04480
EPM Open Late	NO Dist to Cntr, Mean	0.09	0.01564
EPM Open Late	NO Dist to Cntr, Total	0.09	0.01050
EPM Open Late	NO Total Dist	-0.09	0.01230
EPM Open Late	OF Cntr Durt	-0.07	0.04993
EPM Open Late	OF Cntr Freq	-0.08	0.02937
EPM Open Late	OF Cntr Late	0.08	0.03238
EPM Open Late	OF Total Dist	-0.11	0.00154
EPM Total Dist	NO Cntr Durt	0.11	0.00274
EPM Total Dist	NO Cntr Late	-0.14	0.00013
EPM Total Dist	NO Dist to Cntr, Mean	-0.15	$< 1e-04$
EPM Total Dist	NO Dist to Cntr, Total	-0.16	$< 1e-04$
EPM Total Dist	OF Cntr Durt	0.10	0.00680
EPM Total Dist	OF Cntr Freq	0.19	$< 1e-04$
EPM Total Dist	OF Dist to Cntr, Mean	-0.12	0.00074
EPM Total Dist	OF Dist to Cntr, Total	-0.15	$< 1e-04$
EPM Total Dist	SI Dist to Obj Zone, Mean	-0.08	0.02491
EPM Total Dist	SI Dist to Obj Zone, Total	-0.09	0.01678
EPM Total Dist	SI in Obj Zone Durt	0.08	0.02862
EPM Total Dist	SI in Obj Zone Freq	0.12	0.00053
EPM Total Dist	SI in Obj Zone Late	-0.08	0.02312
EPM Total Dist	SI in Soc Zone Freq	0.14	$< 1e-04$

Table S10. Cross behavioral tests correlations, $r < 0.2$, cont'd

Var1	Var2	r	p
MB Num Left	NO Cntr Durt	0.08	0.02911
MB Num Left	OF Cntr Late	-0.07	0.04242
MB Num Left	OF Dist to Cntr, Mean	-0.10	0.00550
MB Num Left	OF Dist to Cntr, Total	-0.10	0.00784
MB Num Left	OF Total Dist	0.09	0.01295
NO Cntr Late	OF Cntr Durt	-0.09	0.00908
NO Cntr Late	OF Cntr Freq	-0.09	0.00998
NO Cntr Late	OF Total Dist	-0.14	0.00012
SI Dist to Obj Zone, Mean	NO Cntr Freq	-0.16	< 1e-04
SI Dist to Obj Zone, Mean	NO Cntr Late	0.11	0.00211
SI Dist to Obj Zone, Mean	NO Total Dist	-0.12	0.00058
SI Dist to Obj Zone, Mean	OF Cntr Durt	-0.13	0.00026
SI Dist to Obj Zone, Mean	OF Cntr Freq	-0.17	< 1e-04
SI Dist to Obj Zone, Mean	OF Dist to Cntr, Total	0.19	< 1e-04
SI Dist to Obj Zone, Mean	OF Total Dist	-0.15	< 1e-04
SI Dist to Obj Zone, Total	NO Cntr Freq	-0.16	< 1e-04
SI Dist to Obj Zone, Total	NO Cntr Late	0.11	0.00259
SI Dist to Obj Zone, Total	NO Total Dist	-0.13	0.00037
SI Dist to Obj Zone, Total	OF Cntr Durt	-0.14	0.00015
SI Dist to Obj Zone, Total	OF Cntr Freq	-0.17	< 1e-04
SI Dist to Obj Zone, Total	OF Dist to Cntr, Total	0.19	< 1e-04
SI Dist to Obj Zone, Total	OF Total Dist	-0.16	< 1e-04
SI Dist to Soc Zone, Mean	NO Cntr Durt	-0.08	0.03521
SI Dist to Soc Zone, Mean	NO Dist to Cntr, Mean	0.09	0.00845
SI Dist to Soc Zone, Mean	NO Dist to Cntr, Total	0.10	0.00805
SI Dist to Soc Zone, Mean	OF Dist to Cntr, Mean	0.09	0.01098
SI Dist to Soc Zone, Mean	OF Dist to Cntr, Total	0.10	0.00557
SI Dist to Soc Zone, Total	NO Cntr Durt	-0.08	0.03672
SI Dist to Soc Zone, Total	NO Dist to Cntr, Mean	0.09	0.01041
SI Dist to Soc Zone, Total	NO Dist to Cntr, Total	0.09	0.00905
SI Dist to Soc Zone, Total	OF Dist to Cntr, Mean	0.09	0.01090
SI Dist to Soc Zone, Total	OF Dist to Cntr, Total	0.10	0.00476
SI in Obj Zone Durt	NO Cntr Freq	0.17	< 1e-04
SI in Obj Zone Durt	NO Dist to Cntr, Mean	-0.18	< 1e-04
SI in Obj Zone Durt	NO Dist to Cntr, Total	-0.17	< 1e-04
SI in Obj Zone Durt	NO Total Dist	0.13	0.00041
SI in Obj Zone Durt	OF Cntr Durt	0.09	0.01782
SI in Obj Zone Durt	OF Cntr Freq	0.14	0.00011
SI in Obj Zone Durt	OF Dist to Cntr, Mean	-0.19	< 1e-04
SI in Obj Zone Durt	OF Dist to Cntr, Total	-0.15	< 1e-04
SI in Obj Zone Durt	OF Total Dist	0.13	0.00025
SI in Obj Zone Freq	NO Cntr Durt	0.08	0.02165
SI in Obj Zone Freq	NO Cntr Late	-0.08	0.03673
SI in Obj Zone Freq	NO Dist to Cntr, Mean	-0.10	0.00754
SI in Obj Zone Freq	NO Dist to Cntr, Total	-0.09	0.00982
SI in Obj Zone Freq	NO Total Dist	0.15	< 1e-04
SI in Obj Zone Freq	OF Cntr Freq	0.17	< 1e-04
SI in Obj Zone Freq	OF Dist to Cntr, Mean	-0.08	0.03328
SI in Obj Zone Freq	OF Total Dist	0.19	< 1e-04
SI in Obj Zone Late	NO Cntr Freq	-0.09	0.01668
SI in Obj Zone Late	NO Dist to Cntr, Mean	0.08	0.02360
SI in Obj Zone Late	NO Dist to Cntr, Total	0.08	0.02009
SI in Obj Zone Late	OF Cntr Durt	-0.07	0.04682

Table S11. Cross behavioral tests correlations, $r < 0.2$, cont'd

Var1	Var2	r	p
SI in Obj Zone Late	OF Dist to Cntr, Mean	0.08	0.02823
SI in Obj Zone Late	OF Dist to Cntr, Total	0.10	0.00672
SI in Obj Zone Late	OF Total Dist	-0.07	0.04419
SI in Soc Zone Durt	NO Cntr Durt	0.12	0.00125
SI in Soc Zone Durt	NO Dist to Cntr, Mean	-0.13	0.00045
SI in Soc Zone Durt	NO Dist to Cntr, Total	-0.13	0.00042
SI in Soc Zone Freq	NO Cntr Freq	0.13	0.00023
SI in Soc Zone Freq	NO Total Dist	0.10	0.00546
SI in Soc Zone Freq	OF Cntr Freq	0.11	0.00222
SI in Soc Zone Freq	OF Dist to Cntr, Total	-0.08	0.02424
SI in Soc Zone Freq	OF Total Dist	0.17	$<$ 1e-04
SI Total Dist	NO Cntr Durt	0.11	0.00160
SI Total Dist	NO Dist to Cntr, Mean	-0.13	0.00039
SI Total Dist	NO Dist to Cntr, Total	-0.14	0.00015
SI Total Dist	OF Cntr Durt	0.13	0.00036
SI Total Dist	OF Dist to Cntr, Mean	-0.11	0.00212
SI Total Dist	OF Dist to Cntr, Total	-0.13	0.00046

Table S12. Principal component regression for nicotine infusion in the first 3 sessions, females

	Estimate	Std. Error	t value	Pr(>|t|)
(Intercept)	3.0253	0.3914	7.73	0.0000
PC3	-0.3905	0.2649	-1.47	0.1414
PC6	0.9865	0.2514	3.92	0.0001
PC7	0.9349	0.4168	2.24	0.0256
PC12	1.2772	0.4000	3.19	0.0016
PC14	-0.4746	0.2999	-1.58	0.1145
PC16	0.6583	0.3807	1.73	0.0847
PC17	-0.9225	0.3903	-2.36	0.0187
PC18	0.6413	0.2893	2.22	0.0274
I(PC7^2)	-0.2180	0.1201	-1.82	0.0704
I(PC12^2)	-0.4553	0.2068	-2.20	0.0284
I(PC16^2)	-0.0869	0.0541	-1.61	0.1092
I(PC17^2)	0.1560	0.1006	1.55	0.1221
I(PC22^2)	0.8948	0.2000	4.47	0.0000

Table S13. Principal component regression for nicotine infusion in the first 3 sessions, males

	Estimate	Std. Error	t value	Pr(>|t|)
(Intercept)	2.4445	0.4127	5.92	0.0000
PC1	-0.4486	0.2413	-1.86	0.0640
PC5	0.7040	0.2365	2.98	0.0031
PC14	-0.5517	0.3712	-1.49	0.1383
PC18	0.5180	0.2317	2.24	0.0261
PC19	0.5377	0.2323	2.31	0.0213
PC21	-0.5101	0.2380	-2.14	0.0330
PC22	0.3650	0.2337	1.56	0.1194
PC23	0.8885	0.3563	2.49	0.0132
PC24	-0.4470	0.2291	-1.95	0.0519
PC25	0.7586	0.2503	3.03	0.0027
PC31	-0.4651	0.3047	-1.53	0.1280
I(PC2ˆ2)	0.4397	0.2728	1.61	0.1081
I(PC13ˆ2)	-0.2949	0.1697	-1.74	0.0833
I(PC14ˆ2)	0.2384	0.1221	1.95	0.0517
I(PC23ˆ2)	-0.1218	0.0747	-1.63	0.1039

Table S14. Principal component regression for nicotine infusion in the last 3 sessions, females

	Estimate	Std. Error	t value	Pr(>|t|)
(Intercept)	4.6773	0.4426	10.57	0.0000
PC5	0.6976	0.3850	1.81	0.0710
PC6	0.5557	0.3321	1.67	0.0954
PC12	1.1969	0.4016	2.98	0.0031
PC19	1.1334	0.3866	2.93	0.0036
PC26	0.5458	0.3870	1.41	0.1595
PC31	-1.1164	0.4555	-2.45	0.0148
I(PC19ˆ2)	-0.2351	0.1394	-1.69	0.0928
I(PC29ˆ2)	-0.2978	0.2093	-1.42	0.1559
I(PC31ˆ2)	0.0934	0.0627	1.49	0.1375

Table S15. Principal component regression for nicotine infusion in the last 3 sessions, males

	Estimate	Std. Error	t value	Pr(>|t|)
(Intercept)	2.1542	0.4248	5.07	0.0000
PC2	-0.7854	0.2872	-2.73	0.0066
PC3	-0.8342	0.3553	-2.35	0.0196
PC5	0.6221	0.2774	2.24	0.0257
PC13	-0.6144	0.2759	-2.23	0.0267
PC15	1.2518	0.3189	3.93	0.0001
PC17	-0.8315	0.3844	-2.16	0.0314
PC21	-0.4452	0.2699	-1.65	0.1001
PC23	0.4191	0.2537	1.65	0.0997
PC25	-0.4489	0.2965	-1.51	0.1312
PC29	0.6096	0.3264	1.87	0.0628
PC30	-0.8838	0.3556	-2.49	0.0135
I(PC1ˆ2)	-0.7279	0.2141	-3.40	0.0008
I(PC3ˆ2)	0.2915	0.1860	1.57	0.1182
I(PC6ˆ2)	0.2867	0.1113	2.58	0.0105
I(PC17ˆ2)	0.1025	0.0650	1.58	0.1161
I(PC20ˆ2)	0.5603	0.1624	3.45	0.0006
I(PC27ˆ2)	0.2436	0.1473	1.65	0.0992

Table S16. Principal component regression for nicotine infusion in the progressive session, females

	Estimate	Std. Error	t value	Pr(>|t|)
(Intercept)	3.5478	0.2766	12.83	0.0000
PC5	-0.5805	0.2517	-2.31	0.0218
PC6	0.5729	0.1626	3.52	0.0005
PC7	0.4275	0.2788	1.53	0.1264
PC9	-0.4031	0.2390	-1.69	0.0928
PC11	0.2623	0.1789	1.47	0.1437
PC12	0.2844	0.1966	1.45	0.1493
PC13	0.3204	0.1793	1.79	0.0750
PC15	-0.3682	0.2065	-1.78	0.0756
PC19	-0.2729	0.1778	-1.53	0.1260
PC20	0.3855	0.2409	1.60	0.1107
PC21	0.3298	0.1878	1.76	0.0801
PC28	-0.2899	0.1945	-1.49	0.1373
I(PC3^2)	-0.1087	0.0735	-1.48	0.1405
I(PC4^2)	-0.0977	0.0448	-2.18	0.0299
I(PC5^2)	0.1963	0.1038	1.89	0.0597
I(PC7^2)	-0.1082	0.0784	-1.38	0.1685
I(PC10^2)	0.1841	0.1047	1.76	0.0798
I(PC18^2)	0.2297	0.1097	2.10	0.0371
I(PC22^2)	0.2584	0.1301	1.99	0.0480
I(PC28^2)	0.0969	0.0509	1.90	0.0581

Table S17. Principal component regression for nicotine infusion in the progressive session, males

	Estimate	Std. Error	t value	Pr(>|t|)
(Intercept)	2.9169	0.2796	10.43	0.0000
PC1	-0.3131	0.1656	-1.89	0.0597
PC5	0.4809	0.2328	2.07	0.0398
PC7	-0.2771	0.1548	-1.79	0.0746
PC13	-0.2872	0.1653	-1.74	0.0833
PC14	-0.4847	0.2511	-1.93	0.0546
PC17	-0.4217	0.2280	-1.85	0.0655
PC18	-0.3108	0.1602	-1.94	0.0534
PC20	0.5847	0.1984	2.95	0.0035
PC21	-0.5527	0.2037	-2.71	0.0071
PC22	0.4586	0.1593	2.88	0.0043
PC23	0.2847	0.1518	1.88	0.0617
I(PC1^2)	-0.2537	0.1031	-2.46	0.0144
I(PC2^2)	0.2378	0.1716	1.39	0.1670
I(PC3^2)	0.1569	0.0871	1.80	0.0729
I(PC5^2)	-0.1937	0.0967	-2.00	0.0461
I(PC14^2)	0.1528	0.0813	1.88	0.0613
I(PC17^2)	0.0742	0.0380	1.95	0.0517
I(PC21^2)	0.1846	0.0699	2.64	0.0087
I(PC31^2)	-0.1820	0.0775	-2.35	0.0195

Table S18. Principal component regression for the number of licks on the active spout in the reinstatement session, females

	Estimate	Std. Error	t value	Pr(>|t|)
(Intercept)	136.2752	8.4385	16.15	0.0000
PC5	-14.3487	8.5780	-1.67	0.0955
PC6	20.0105	5.6241	3.56	0.0004
PC8	18.1113	10.4151	1.74	0.0831
PC9	-13.9640	8.2100	-1.70	0.0901
PC11	8.6049	6.1446	1.40	0.1625
PC15	-10.2034	7.0028	-1.46	0.1462
PC20	13.6462	7.6545	1.78	0.0757
I(PC5^2)	5.2851	3.6073	1.47	0.1440
I(PC8^2)	-2.1791	1.2422	-1.75	0.0805
I(PC19^2)	-4.7004	2.1707	-2.17	0.0312
I(PC29^2)	-5.6583	3.4627	-1.63	0.1034

Table S19. Principal component regression for the number of licks on the active spout in the reinstatement session, males

	Estimate	Std. Error	t value	Pr(>|t|)
(Intercept)	85.9835	8.8945	9.67	0.0000
PC1	-10.4452	5.6588	-1.85	0.0660
PC5	16.1508	7.9582	2.03	0.0434
PC7	-9.2312	5.2618	-1.75	0.0805
PC13	-8.4289	5.6518	-1.49	0.1370
PC14	-17.5476	8.5678	-2.05	0.0415
PC17	-14.0426	7.7806	-1.80	0.0722
PC18	-11.0610	6.0756	-1.82	0.0698
PC20	20.2899	6.6242	3.06	0.0024
PC21	-15.1549	6.9248	-2.19	0.0295
PC23	11.0761	5.1430	2.15	0.0321
I(PC1^2)	-6.1825	3.4986	-1.77	0.0783
I(PC5^2)	-6.0484	3.1759	-1.90	0.0579
I(PC6^2)	3.4358	2.1888	1.57	0.1176
I(PC14^2)	5.3925	2.7909	1.93	0.0544
I(PC17^2)	3.2168	1.2963	2.48	0.0137
I(PC18^2)	-3.6843	2.4767	-1.49	0.1380
I(PC21^2)	6.8734	2.3868	2.88	0.0043
I(PC27^2)	4.5164	2.9586	1.53	0.1280
I(PC31^2)	-4.0759	2.5316	-1.61	0.1085
